# Image classification of osteoporotic vertebral fracture with endplate-disc complex Injury

**DOI:** 10.1186/s12891-021-04070-6

**Published:** 2021-02-17

**Authors:** Shuai Zhang, Song Wang, Qing Wang, Jin Yang, Shuang Xu

**Affiliations:** Department of Orthopedics, The Affiliated Hospital of Southwest Medical University, NO. 25 Taiping S tr eet, Sichuan 646000 Luzhou City, China

**Keywords:** Classification, Computerized tomography, Endplate-disc complex injury, Magnetic resonance imaging, Osteoporotic vertebral fractures

## Abstract

**Background:**

The purpose of this study was to use MRI and CT to observe osteoporosis vertebral fracture (OVF) combined with endplate-disc complex (EDC) injury and to classify the degree of EDC injury according to the changes in EDC signal intensity and morphology on the images.

**Methods:**

We investigated the incidence of EDC injury, observed the morphology and signal intensity changes of EDC injury using MRI and CT, and graded the injuries from 0 to 4 according to their severity. We compared whether there were differences in the degree of EDC injury among different vertebral fractures, bone mineral density(BMD), and severity of vertebral fractures.

**Results:**

A total of 479 patients were included in this study, of whom 321 had EDC injury adjacent to the fractured vertebral body. Among those, 158 cases were grade 0, 66 cases were grade 1, 72 cases were grade 2, 78 cases were grade 3, and 92 cases were grade 4. The degree of EDC injury associated with thoracolumbar vertebral fractures was more serious than that of EDC injuries associated with thoracic and lumbar vertebral body fractures. Vertebral fractures with severe osteoporosis were associated with more severe EDC injury. Additionally, the more severe the vertebral fracture, the more severe was the combined EDC injury.

**Conclusion:**

This study found that the incidence rate of EDC injury reached 67.0%. Among patients with OVF, severe osteoporosis and severe fractures in the thoracolumbar segments were often associated with more severe EDC injury.

## Background

With the increased aging of the global population, osteoporosis is threatening greater numbers of people or is rising in frequency. Osteoporotic vertebral fracture (OVF) is one of the most common sequelae of osteoporosis. It often leads to lower back pain and spinal deformity, which seriously affects the patients’ quality of life [1–3]. In order to increase the patient’s ability to perform daily activities and relieve pain, braces can usually be used to protect the spine, exercises can be done to strengthen core muscles, and patients can take oral non-steroidal anti-inflammatory analgesics or opioid analgesics [4–6]. In order to delay the progress of osteoporosis and improve bone metabolism, some studies have reported that subcutaneous injection of denosumab or teriparatide and standardized oral bisphosphonates can achieve satisfactory clinical effects [7, 8]. Percutaneous kyphoplasty (PKP) is a good treatment for patients with OVF who cannot bear the pain or do not get well from conservative treatment [9–12]. However, the operation is focused mainly on the fractured vertebral body itself, ignoring the treatment of adjacent injured endplate-disc complex (EDC) [13, 14]. The EDC has important functions such as maintaining the stability and integrity of the spine, protecting the spinal nerves, absorbing shocks, and dispersing axial load. When the trauma acts on the vertebral body, it often causes injury to the adjacent EDC at the same time. Injuries to EDCs, which are the human body’s largest blood-free tissue, often accelerate disc degeneration, leading to chronic spinal instability and even secondary kyphosis [15–17]. Sander et al. [18] classified vertebral body fracture combined with intervertebral disc (IVD) injury based on magnetic resonance imaging (MRI) changes of IVD morphology and signal intensity. However, their small sample did not include OVF combined with IVD injury, so their classification is not applicable to the combined injuries. Fujiwara et al. [19] observed IVD injury in patients with OVF graded according to the Sander et al. classification; however, the injury mechanism and degree of IVD injury in OVF may be different than in those with normal bone mass, and thus unsuitable for classification of EDC injury combined with OVF. Ghanem et al. [20] found that MRI is an effective non-invasive method for diagnosing IVD injury. Although MRI is more sensitive to signal intensity changes caused by IVD injury, it is less sensitive to endplate fractures. Because the endplate is an important part of the EDC, its treatment is as important as the treatment of vertebral fractures. Computed tomography (CT) multiplanar reconstruction (MPR) technology can clearly reveal endplate injury. The main purpose of this study was to combine MRI and CT to observe the condition of OVF patients with EDC injury, performing image classification according to the degree of injury, in order to improve communication between spine surgeons and radiologists as well as to facilitate clinical decision-making in spine surgery.

### Methods

 The study was authorized by the Ethics Committee of the Affiliated Hospital of Southwest Medical University.

### Patient Population

From August 2017 to August 2020, patients diagnosed with an acute OVF of thoracic or lumbar vertebrae were reviewed at the orthopedics department of the Affiliated Hospital of Southwest Medical University. All data were retrospectively reviewed based on medical records and billing statements. All patients had standard thoracolumbar anterior and lateral plain radiographs (Ziehm Solo, Ziehm imagine GMBH, Germany), plain CT scan and MPR images of the fracture level (LightSpeed VCT, GE Healthcare, IN), and MRI of the whole spine (Signa HDe, GE Healthcare Japan, Tokyo). The inclusion criteria were as follows: (1) clinical manifestations of waist and back pain, with pain aggravated by turning over or getting up; (2) no signs or symptoms of spinal cord or nerve root damage in the corresponding fracture segment; (3) presence of osteoporosis as determined by dual energy X-ray absorptiometry; (4) and single vertebral body fractures on MRI with low signal intensity on T1-weighted imaging, high signal intensity on T2-weighted imaging, and high signal intensity on short TI inversion recovery (STIR). The exclusion criteria were as follows: (1) serious spinal instability caused by pedicle fracture; (2) symptomatic neurologic injury; (3) and non-OVF conditions, such as tumors or infectious diseases, that were confirmed by pathological examination.

### Image classification of EDC injury in OVF patients

**Fig. 1:**
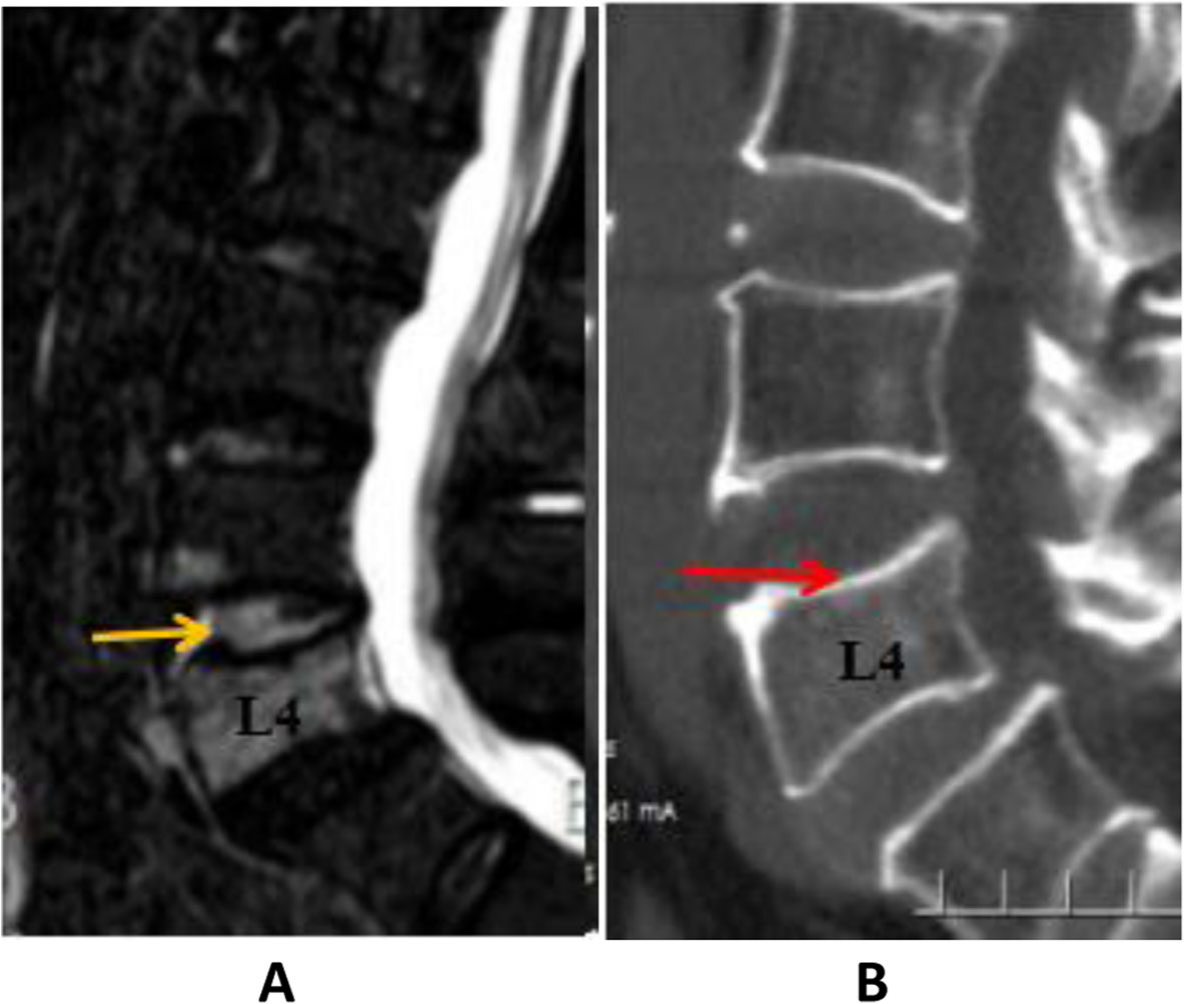
A 68-year-old man with OVF at level L4. Panel **a** is an MRI STIR image. The yellow arrow indicates a diffuse high-intensity signal in the IVD on the cranial side of the fractured vertebral body whose signal intensity is close to that of cerebrospinal fluid. Panel **b** is a CT image of the fractured vertebral body. The red arrow indicates that the upper endplate of the L4 vertebral body is intact. This is a grade 1 EDC injury as delineated in this study

**Fig. 2:**
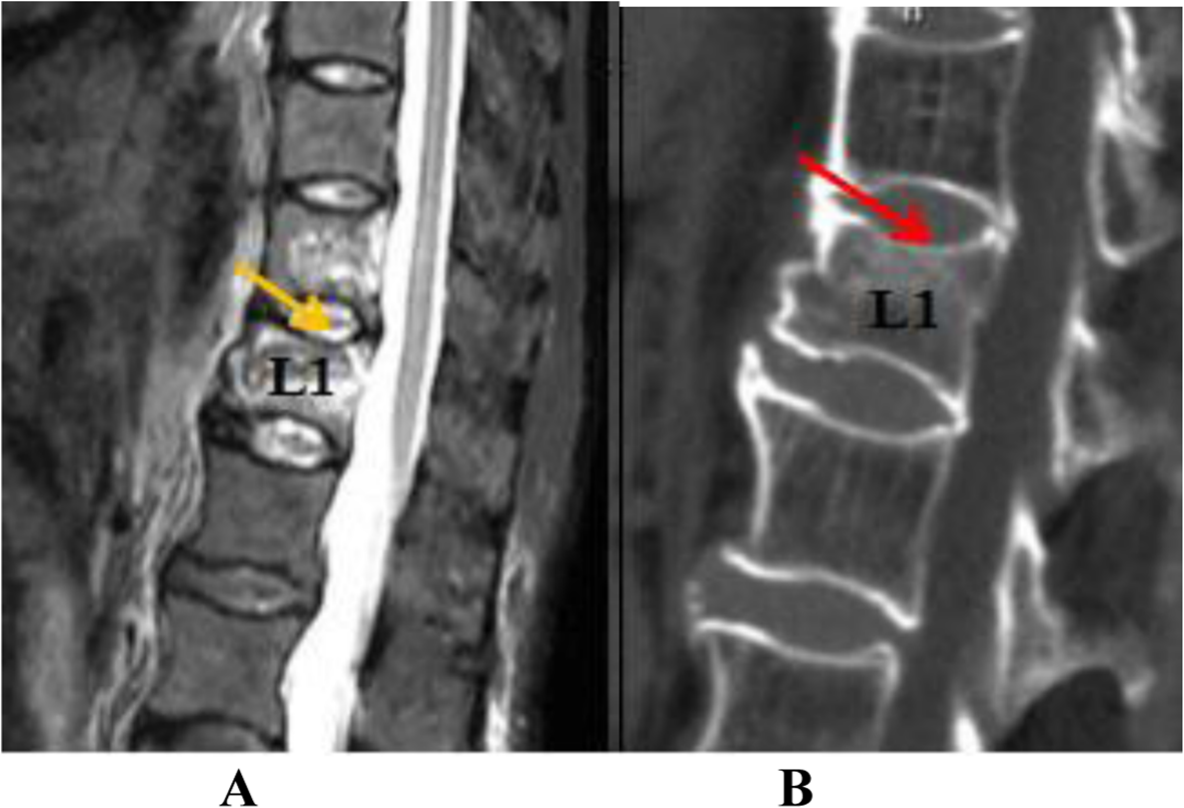
A 73-year-old woman with OVF at level L1. Panel **a** is an MRI STIR image. The yellow arrow indicates a diffuse high signal in the IVD on the cranial side of the fractured vertebral body. Panel **b** is a CT image of the fractured vertebral body. The red arrow indicates a linear fracture of the upper endplate of the L1 vertebral body, but there is no sign of displacement or collapse of the endplate. This is a grade 2 EDC injury as delineated in this study

**Fig. 3:**
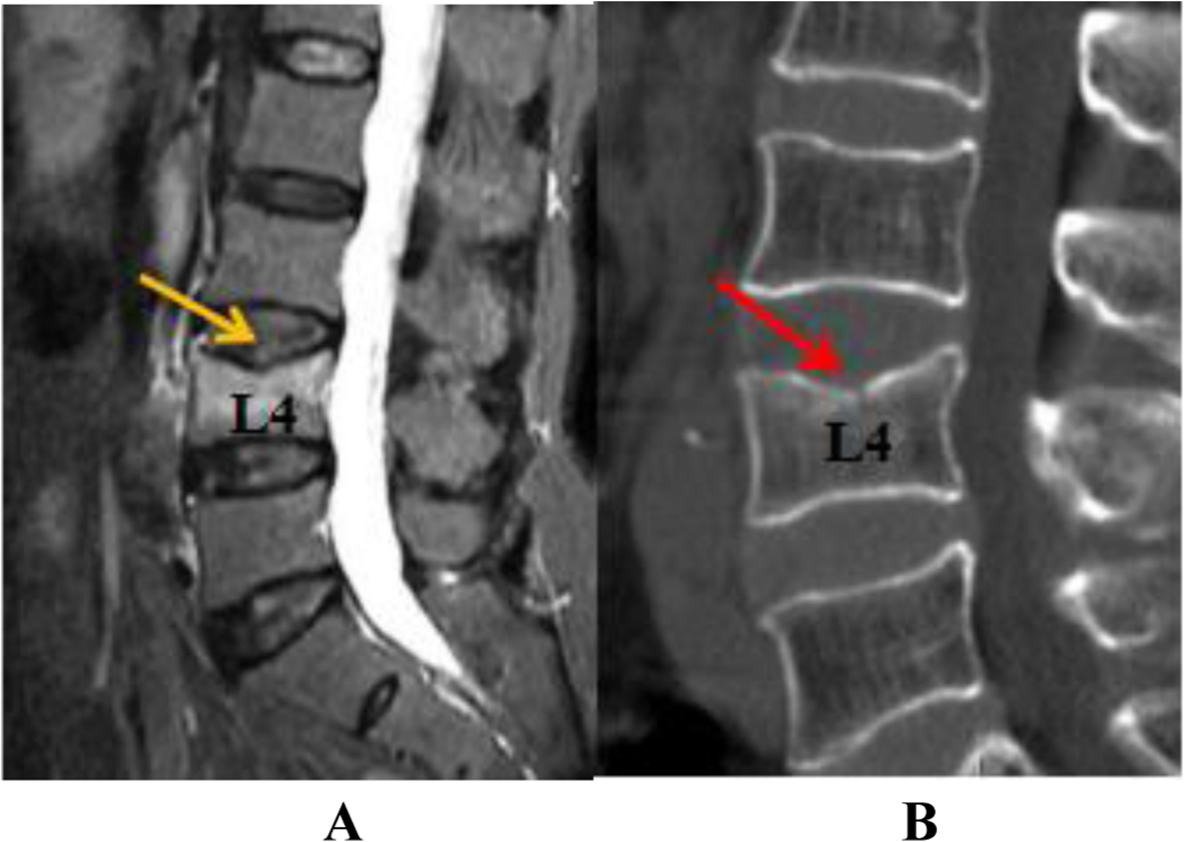
A 76-year-old woman with OVF at level L4. Panel **a** is an MRI STIR image. The yellow arrow indicates a diffuse high signal in the OVF on the cranial side of the fractured vertebral body and a fracture of the upper endplate of the vertebral body. Panel **b** is a CT image of the fractured vertebral body. The red arrow indicates a fracture of the upper endplate of the L4 vertebral body. The endplate has collapsed with signs of bone sclerosis below the endplate. This is a grade 3 EDC injury as delineated in this study

**Fig. 4:**
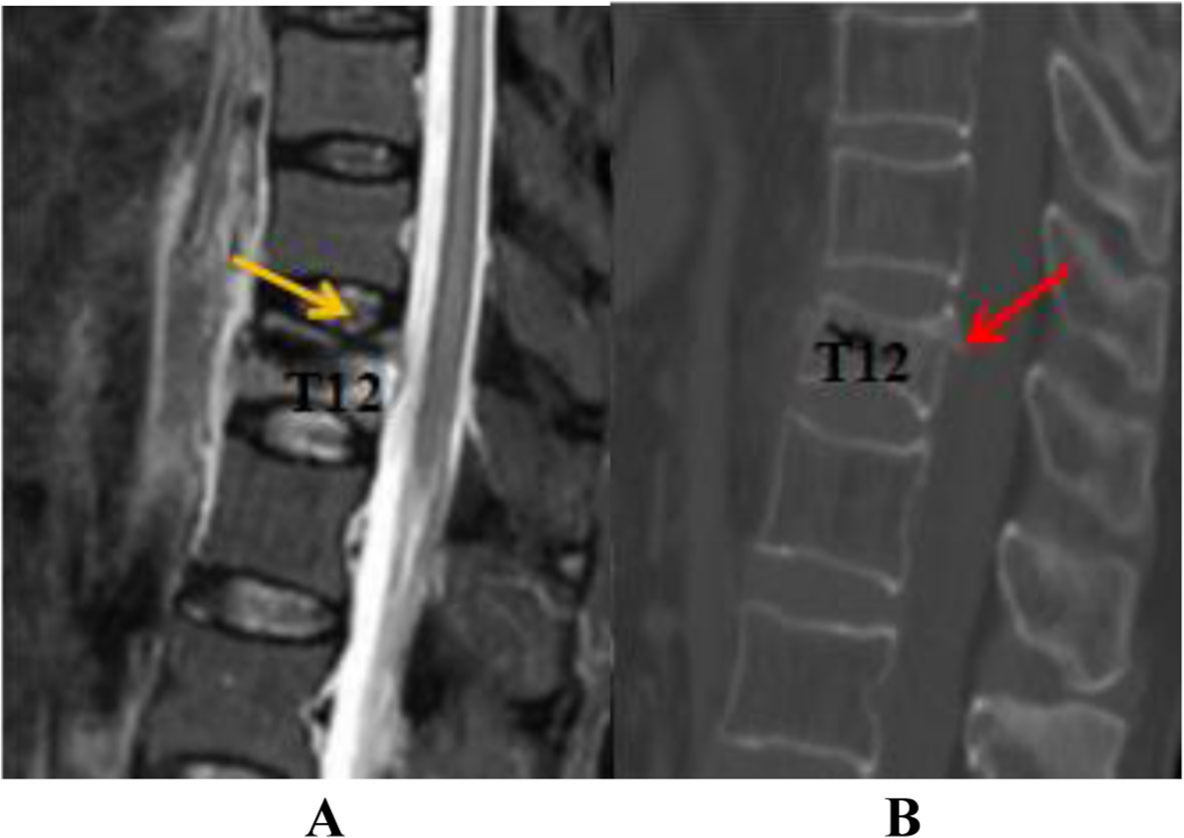
A 74-year-old woman with OVF at level T12. Panel **a** is an MRI STIR image. The yellow arrow indicates a diffuse high signal in the IVD on the cranial of the fractured vertebral body and a fracture of the upper endplate of the vertebral body. Figure **b** is a CT image of the fractured vertebral body. The red arrow indicates that the upper endplate of the T12 vertebral body is fractured, and the endplate collapse is accompanied by a fracture of the posterior vertebral body. This is a grade 4 EDC injury as delineated in this study

To differentiate acute OVF from vertebral body fat deposits, chronic OVF, vertebral body hemangioma, and other signs, this study used MRI STIR imaging combined with CT MPR technology to diagnose acute OVF and combined EDC injury. According to the changes of the morphology and signal intensity of the IVD adjacent to the OVF on the STIR image together with the endplate injury on the CT reconstructed image, EDC injury combined with OVF was divided into grades 0–4(Figs. 1, 2, 3 and 4). Grade 0: STIR image and CT reconstruction observation revealed that the morphology and signal intensity of the EDC were normal compared with the uninjured EDC in the distant part, indicating that the EDC was not injured. Grade 1: STIR image shows diffuse or localized high signal intensity in the IVD, and no endplate fracture signs are found in the CT reconstruction image; this represents intradiscal edema or hemorrhage. Grade 2: STIR image shows diffuse or localized high signal intensity in the IVD, the CT reconstruction image reveals a linear fracture of the endplate, with no displacement or collapse of the endplate and no subendplate sclerosis or subendplate effusion; this represents EDC injury. Grade 3: In addition to grade 2 changes, CT reconstruction images show endplate displacement, collapse, subendplate bone sclerosis, subendplate effusion, and even part of the IVD herniated into the vertebral body. This type of EDC injury is more serious. Grade 4: Grade 2 or 3 changes complicated with posterior wall fracture.

### Intra- and inter‐observer agreement assessment of EDC injury

One orthopedic surgeon evaluated the images twice to assess intra-observer agreement. To assess inter-observer agreement, two orthopedic surgeons evaluated 50 randomly selected patients’ vertebrae. Intra- and interobserver agreements were assessed by calculating the Cohen kappa coefficient. A kappa of < 0.00 was interpreted as minimal agreement, 0.00-0.20 as slight agreement, 0.21–0.40 as fair agreement, 0.41–0.60 as moderate agreement, and 0.61 or over as substantial agreement.

### Data analysis

According to fracture level, OVFs were divided into thoracic (T6-T10), thoracolumbar (T11-L2), and lumbar (L3-L5) groups. For each group, we compared the incidence and degree of EDC injury. According to the diagnostic criteria recommended by the WHO, the included cases were divided into an osteoporosis group (-3.5 < T score ≤ -2.5) and a severe osteoporosis group (T score ≤ -3.5), and the incidence and degree of injury of the two groups of patients with EDC injury were compared. Following the Genant [18] semi-quantitative method for grading vertebral fractures, OVF was graded 0–3, and the incidence of different fractures combined with EDC injury and the degree of injury were compared.

### Statistical analysis

Statistical analysis was performed using the commercial software package SPSS 19.0 (SPSS, Chicago, Illinois, USA). All results for continuous variables are presented as mean ± SD, and those for categorical variables are expressed as n. The Kruskal-Wallis H rank-sum test was used to compare the difference in the incidence and degree of injury between different fracture sites and different fracture levels combined with EDC injury. The Wilcoxon rank-sum test was used to compare the difference in the incidence and degree of injury between osteoporosis patients and severe osteoporosis patients with EDC injury. *P* < 0.01 was taken to indicate statistically significant differences.

## Results

A total of 1249 cases of OVF were diagnosed in the orthopedics department of the Affiliated Hospital of Southwest Medical University. Of these, 479 cases met our inclusion criteria; 321 cases were combined with EDC injury, with 308 cases involving the cranial disc and 13 cases the caudal disc. (Due to the low incidence of caudal disc injury, this study did not include it in the scope of observation.) There were 120 male and 359 female patients; patient ages ranged from 57 to 92 (71.8 ± 6.9) years old, with 108 cases of same-level fall injuries, 96 cases of fall from a height, 133 cases of waist sprain, and 142 cases of no clear history of trauma. Intra- and inter-observer agreements for the assessment of EDC injury were both “substantial,” with kappa values of 0.86 and 0.82, respectively.

In the thoracic group (T6-T10), there were 143 cases of OVF combined with EDC injury that we assigned combined grades as follows: 78 cases of grade 0, 27 cases of grade 1, 18 cases of grade 2, 13 cases of grade 3, and 7 cases of grade 4. In the thoracolumbar group (T11-L2), there were 218 cases of OVF combined with EDC injury as follows: 34 cases of grade 0, 19 cases of grade 1, 37 cases of grade 2, 53 cases of grade 3, and 75 cases of grade 4. In the lumbar group (L3-L5), there were 105 cases of OVF combined with EDC injury as follows: 46 cases of grade 0, 20 cases of grade 1, 17 cases of grade 2, 12 cases of grade 3, and 10 cases of grade 4. The incidence of OVF combined with EDC injury was highest in the thoracolumbar group, and the degree of damage was severe. Compared with the thoracic group and the lumbar group, the differences were statistically significant (*P* < 0.01) (Table 1).

**Table 1:** Comparison of the incidence and degree of injury of OVF combined with EDC injury in different fracture levels

	Grade [cases(%)]				
Fracture level(n)	0	1	2	3	4
Thoracic group(143)	78(54.5)	27(18.9)	18(12.6)	13(9.1)	7(4.9)
Thoracolumbar group(218)	34(15.6)	19(8.7)	37(17.0)	53(24.3)	75(34.4)
Lumbar group(105)	46(43.8)	20(19.1)	17(16.2)	12(11.4)	10(9.5)
χ^2^	107.427				
P	0.000				

There were 246 cases in which the bone mineral density (BMD) T score was below − 3.5, with EDC injury grades as follows: 49 cases of grade 0, 23 cases of grade 1, 43 cases of grade 2, 57 cases of grade 3, and 74 cases of grade 4. There were 220 cases in which the BMD T score was between − 3.5 and − 2.5, with EDC injury grades as follows: 109 cases of grade 0, 43 cases of grade 1, 29 cases of grade 2, 21 cases of grade 3, and 18 cases of grade 4. The incidence of severe osteoporosis combined with EDC injury is higher, and the degree of injury is severe. Compared with the osteoporosis group, the differences were statistically significant (*P* < 0.01) (Table 2).

**Table 2:** Comparison of the incidence and degree of injury of OVF combined with EDC injury in patients with osteoporosis and severe osteoporosis

	Grade [cases(%)]				
The T score of BMD	0	1	2	3	4
-3.5 < T score≤-2.5	109(49.5)	43(19.5)	29(13.2)	21(9.5)	18(8.3)
T score≤-3.5	49(19.9)	23(9.3)	43(17.5)	57(23.2)	74(30.1)
Z	-8.733				
P	0.000				

Using the Genant et al. [21] semi-quantitative method, there were 101 cases of grade 0 OVF, with the distribution of EDC injuries being 74 grade 0, 4 grade 1, 4 grade 2, 10 grade 3, and 9 grade 4. Among the 83 cases of grade 1 OVF, the EDC injury distribution was 30 grade 0, 15 grade 1, 11 grade 2, 13 grade 3, and 14 grade 4. Among the 132 cases with grade 2 OVF, the distribution of EDC injury severity was 33 grade 0, 36 grade 1, 20 grade 2, 21 grade 3, and 22 grade 4. Finally, among the 150 cases of grade 3 OVF, the EDC injury distribution was 21 grade 0, 11 grade 1, 37 grade 2, 34 grade 3, and 47 grade 4. The more severe the OVF injury, the higher the incidence of combined EDC injury, and the greater the severity of the EDC injury. There were statistically significant differences between groups of different fracture degrees (*P* < 0.01) (Table 3).

**Table 3:** Comparison of the incidence and severity of EDC injury associated with different degrees of vertebral body fractures

	Grade [cases(%)]				
The severity of vertebral fracture(n)	0	1	2	3	4
Genant Grade 0 (101)	74(73.3)	4(4.0)	4(4.0)	10(9.9)	9(8.8)
Genant Grade 1 (83)	30(36.1)	15(18.1)	11(13.3)	13(15.7)	14(16.8)
Genant Grade 2 (132)	33(25.0)	36(27.3)	20(15.2)	21(15.9)	22(16.6)
Genant Grade 3 (150)	21(14.0)	11(7.3)	37(24.7)	34(22.7)	47(31.3)
c^2^	80.796				
P	0.000				

## Discussion

PKP surgical treatment of OVF can immediately relieve the patient’s pain, restore the height of the vertebral body, and correct the kyphosis, so it has gradually become one of the main methods for OVF treatment [9–12]. However, some patients still have local pain after the operation, complicated by adjacent vertebral fractures, secondary local instability, and even local kyphotic deformity [13, 14]. Takahashi et al. [22] reported that the above-mentioned complications were mainly caused by PKP surgery being focused solely on the fractured vertebral body itself, ignoring the treatment of the adjacent EDC injury. As the degeneration of the injured IVD accelerates, the local stability of the fracture is lost, and the uneven axial load distribution will eventually result in local chronic pain, adjacent vertebral fractures, and even progressive loss of correction in severe cases. Lin et al. [23] have shown that 60 % of the instability caused by spinal fractures arises from the EDC. Boeree et al. [24] proposed that the integrity of the IVD above the fractured vertebral body is an important factor in maintaining stability.

Although the EDC is an important structure for maintaining the integrity of the spine, dispersing axial load, and absorbing shock, there are very few studies of OVF combined with EDC injury. Because the MRI technology represented by diffusion tensor imaging has extremely high sensitivity in diagnosing acute spinal fractures[25]. Sander et al. [18] first classified the injuries of the intervertebral discs based on the MRI images of spinal fractures. However, the study included patients with normal bone, and its classification method is not completely applicable to patients with osteoporosis. Ortiz et al. [26] were the first to report the incidence of OVF patients having IVD injury and its impact on the treatment effect, but they did not classify and observe the degree of IVD injury associated with OVF. Takahashi et al. [22] conducted research primarily on whether the healing of OVF in patients with IVD injuries depended on the Sander et al. classification. At present, there is no relevant research on the classification of OVF combined with EDC injury. Moreover, separate MRI examinations were used in the past to observe whether the intervertebral disc was damaged, but damage of the endplate, which is an important part of the intervertebral disc, was not mentioned. This study used both MRI and CT to observe OVF with combined EDC injury for the first time.

Conventional wisdom holds that the trauma causing OVF is usually minor, and that it is not easy to damage adjacent structures [27, 28]. However, the rate of OVF combined with IVD damage in this study was as high as 67.0 %, which is slightly higher than the results of Ortiz and Takahashi [22, 26]. This may be because this study used the highly sensitive STIR sequence to observe changes of the IVD signal, in combination with CT MPR technology, to improve the rate of accurate diagnosis of EDC injury. This study found that the incidence of EDC injury was higher, and the degree of injury more severe, for patients in the thoracolumbar group than for those in the thoracic and lumbar groups. The main reasons are as follows: (1) the thoracolumbar vertebral body is located at the junction of thoracic kyphosis and lumbar lordosis; (2) the thoracolumbar vertebral body is in the stress junction area between the thoracic cage and the lumbar spine; and (3) the thoracolumbar vertebral body does not have the thoracic cage and strong psoas major muscle protection. Once it is traumatized, the thoracolumbar vertebral body is prone to fractures, and the degree of fracture and the combined degree of EDC damage are often very serious. This study found that OVF in patients with severe osteoporosis was associated with a higher incidence and severity of EDC injury, which may be principally because patients with severe osteoporosis have greater bone loss, and consequently their vertebral bodies become more fragile. Once the vertebral body fractures, it is easier to spread to the vertebral body endplates and result in IVD damage. The IVD and the adjacent vertebral body are the integral movement unit. Whether acting on the IVD or the vertebral body, destructive force can easily be transmitted to the adjacent structure and cause damage. Employing the Genant et al. [21] semi-quantitative method, this study found that the degree of vertebral body fracture is almost the same as the degree of EDC injury. The more serious the degree of vertebral body fracture, the higher the incidence of combined EDC injury and the more severe the degree of EDC injury.

Generally, conservative treatment of OVF is the first choice, and bed rest, brace protection, orally non-steroidal anti-inflammatory analgesics or opioid analgesics, and even subcutaneous injection of denosumab or teriparatide can achieve satisfactory clinical effects [4–8]. PKP is a good treatment for patients of OVF who cannot bear the pain or do not get well from conservative treatment, and can quickly relieve acute pain and obtain satisfactory clinical effects [9–12]. However, secondary degeneration of the injured intervertebral disc, local instability, adjacent vertebral body fractures, and secondary kyphosis will significantly affect the patient, and may even require repeat surgical treatment. Such patients are often elderly, often with multiple organ dysfunction, and it may be difficult for them to tolerate reoperation. Therefore, the first treatment for OVF and the combined injury of the EDC is very important. According to our team’s experience, if the EDC injury is grade 0 or 1, we recommend that the treatment be selected based on the degree of vertebral fracture without special treatment of the injured IVD. If the EDC injury is grade 2, we recommend PKP to treat OVF as the first choice. In addition, intraoperative subendplate distribution of bone cement should be achieved as much as possible to prevent the damaged endplate from collapsing. If the EDC injury is classified as grade 3 or 4 and the patient can tolerate surgery, then discectomy, reduction of the fractured vertebral body and injured endplate, intervertebral bone grafting, and internal fixation are preferred for the patients. If the patient cannot withstand general anesthesia, percutaneous vertebral body-intervertebral disc cementoplasty may also be an effective treatment methods [29]. However, when injecting bone cement, it is necessary to prevent the bone cement from leaking into the spinal canal through the fracture line of the posterior wall of the vertebral body. Certainly, the above treatment methods need to be combined with standard anti-osteoporosis treatment, brace protection, and enhancement of core muscle strength. As one of the main structures of the integral movement unit, the EDC plays a vital role in maintaining the stability and integrity of the spine and in protecting the nerves and dispersing the axial load. Especially for OVF patients, there may only be one chance for surgery. Clinicians can carefully screen OVF combined with EDC injuries according to the classification criteria formulated herein and choose individual treatment methods to maximize the therapeutic effect of OVF.

There are two principal limitations to this study. First, this was a retrospective observational study in which patients had variations in bone mineral density, past OVF history, pharmaceutical agent use, duration between onset and hospital admission, and timing of MRI examination with a potential selection bias. A second limitation was the small sample size. To gain greater reliability for guiding the choice of treatment will require long-term clinical observation and follow-up.

## Conclusions

The incidence of OVF combined with EDC injury is 67.0 %, and the cranial side of the fractured vertebral body is more likely to be involved. Combining MRI and CT to observe the morphology and signal changes of adjacent EDC in patients with OVF, the combination of OVF and EDC injury can be divided into five grades from 0 to 4, with incidence rates of 33.9 %, 14.2 %, 15.5 %, 16.7 %, and 19.7 %, respectively. In patients with OVF, both severe osteoporosis and severe fractures in the thoracolumbar segments are often combined with more severe EDC injury. Further studies must be conducted to verify clinical relevance.

## Data Availability

Data will be available upon request to the first author ZS.

## Abbreviations

BMD: Bone mineral density
CT: Computed tomography
EDC: Endplate-disc complex
IVD: Intervertebral disc
MPR: Multiplanar reconstruction
MRI: Magnetic resonance imaging
OVF: Osteoporotic vertebral fracture
PKP: Percutaneous kyphoplasty
STIR: Short TI inversion recovery
